# Reimbursement of Orphan Drugs in Europe in Relation to the Type of Authorization by the European Medicines Agency and the Decision Making Based on Health Technology Assessment

**DOI:** 10.3389/fphar.2018.01263

**Published:** 2018-11-12

**Authors:** Krzysztof Piotr Malinowski, Paweł Kawalec, Wojciech Trabka, Christoph Sowada, Andrzej Pilc

**Affiliations:** ^1^Faculty of Health Sciences, Institute of Public Health, Jagiellonian University Medical College, Krakow, Poland; ^2^Bioinformatics and Public Health Department, Faculty of Medicine and Health Sciences, Andrzej Frycz Modrzewski Krakow University, Krakow, Poland; ^3^Institute of Pharmacology, Polish Academy of Sciences, Krakow, Poland

**Keywords:** EMA (European Medicines Agency), orphan drugs for rare diseases, reimbursement, authorization, HTA (Health Technology Assessment)

## Abstract

**Objective:** To assess shares of reimbursed orphan drugs and agreement in reimbursement decision-making in different European Union member states as well as to define odds for reimbursement influenced by the presence of conditional approval or exceptional circumstances granted by the European Medicines Agency (EMA) or by type of the disease.

**Methods:** The list of authorized drugs with current orphan designations was collected from the website of the EMA. For each drug, the information regarding conditional approval or approval under exceptional circumstances was collected. The reimbursement statuses were available on national reimbursement or HTA agencies websites. The agreement for reimbursement decisions between selected countries was assessed using the κ coefficient for the measurement of agreement. The impact of the EMA's conditional approval as well as approval under exceptional circumstances was assessed using the logistic regression and presented as odds ratio.

**Results:** The percentage of reimbursed orphan drugs varied significantly from 27% in Poland to 88% in Denmark, with an average value of 51% (*p* < 0.0001). Regarding the reimbursement status, the highest, substantial agreement was observed between Spain and Italy, and the lowest agreement was observed between Germany and England, with κ of 0.64 and 0.01, respectively. Conditional approval status significantly decreased the chance for reimbursement in France, Italy, and Spain by 77–80%; however, approval granted under exceptional circumstances had significant impact only in Germany with 85% decrease in chances for reimbursement. The type of the disease (oncology or metabolic) was significantly associated with both conditional approval (*p* of 0.03—oncology drugs were more likely to be conditionally approved then the rest of analyzed drugs) and exceptional circumstances (*p* of 0.02—drugs for metabolic diseases were more likely to be approved under exceptional circumstances).

**Conclusions:** Access to reimbursed orphan drugs varies significantly across EU countries. The highest, substantial agreement in reimbursement decisions was observed between Italy and Spain and the lowest between Germany and England. Conditional approval and approval under exceptional circumstances were significant negative predictors of reimbursement in some countries and they were significantly associated with the type of the disease (oncology or metabolic).

## Background

There is no common definition of an orphan drug, which is the reason for discrepancies among the definitions implemented by different countries in their drug reimbursement decision-making process. However, there is general acceptance that the definition should be based on the prevalence of rare diseases treated by orphan drugs. According to the current definition provided by the European Union (EU), rare diseases mostly include inherited, life-threatening, or chronically debilitating diseases that affect fewer than 5 out of 10,000 people (EMEA, 2017). The assumed threshold prevalence varied from 1 to 8 per 10,000 people (Winstone et al., 2015). According to the definition by the European Medicines Agency's (EMA), the prevalence is 5 persons per 10,000, which translates into around 246,000 people affected by rare diseases, considering 27 EU member states (Winstone et al., 2015; EMEA, 2017).

Different types of rare diseases can be defined and the broadest categories include oncologic diseases (around 32.5% of all orphan drugs; Gammie et al., 2015) and metabolic conditions. As orphan diseases have mostly genetic origin both oncologic and metabolic orphan drugs are of special interest for EMA.

In order for a drug to fulfill the EMA's conditions of orphan drugs, it needs to be used for the diagnosis, prevention, or treatment of patients with a life-threatening or chronically debilitating condition. The EMA's definition includes also drugs that are unlikely to generate sufficient profit to justify research and development costs (Winstone et al., 2015; EMEA, 2017). This doubtful and uncertain return on the investment makes the health technology assessment (HTA) process very difficult and challenging because the required data on clinical efficacy and safety as well as data pertaining to costs may simply be insufficient. What makes the reimbursement decision even harder is the fact that orphan drugs are generally more expensive than non-orphan drugs due to frequent genetic etiology of the targeted disease (EMEA, 2017), which translates into significant budget impact despite a low number of potential patients. It is an important aspect of proper allocation of public finances presenting a major problem for public health and decision-making. This fact may be reflected in substantial variation of reimbursement decisions for orphan drugs among EU state members.

Orphanet (https://www.orpha.net/consor/cgi-bin/index.php) is a reference portal with information on rare diseases and orphan drugs for all audiences. Its goal is to gather and improve knowledge on rare diseases, their diagnosis, and nomenclature as well as care and treatment of patients with these diseases (Orphanet, 2018).

To help national decision-makers as well as national HTA agencies, the EMA issues a conditional marketing authorization (also known as a conditional approval) indicating that the medicine is addressed to fulfill important and unmet treatment needs of patients (which is often the case in patients with rare diseases). The data for the approval are less comprehensive than normally required. The presented data, however, are demanded to indicate that potential benefits from applying the treatment are higher than potential losses (risks). The marketing authorization holder is then obligated to provide a comprehensive body of clinical evidence in the future, usually within a time frame negotiated with the EMA. This conditional approval could be a signal for national decision-makers that comprehensive data will be available (EMA, 2017).

However, in some cases the condition to be treated is rare or the collection of detailed information is impossible or unethical. In these situations, the EMA may grant a marketing authorization in absence of comprehensive data under exceptional circumstances. It is a type of marketing authorization granted to medicines of which the marketing authorization holder is unable (and will probably never be able) to provide comprehensive data on the efficacy and safety under normal conditions of use.

Unlike conditional marketing authorization, in which marketing approval is granted in the likelihood that the sponsor will provide such data within an agreed time frame, authorization under exceptional circumstances can be granted when comprehensive data cannot be obtained even after authorization. This could be an important signal for national decision-makers and could potentially influence their decision, especially in a situation of a very limited budget (Commission Regulation, 2006).

Our objective was to assess the share of reimbursed orphan drugs as well as the agreement in reimbursement decision-making in different EU member states; we would like to evaluate if reimbursement decisions are influenced by the presence of conditional approval or exceptional circumstances granted by EMA. In addition the impact of type of disease (oncologic or metabolic) on conditional approval and approval under exceptional circumstances was examined.

## Methods

The list of authorized drugs with current orphan designations was collected from the EMA website (on 24 January 2017) (EMA, 2018). A list of countries that had databases of reimbursed drugs publicly available and that allowed for such an analysis to be performed was composed. The reimbursement status of each drug was collected for Belgium, Denmark, England, France, Germany, Italy, Poland, Scotland, Spain, Sweden, The Netherlands, and Wales (Tables 1, 2). To perform sophisticated and in-depth analysis, data on recommendations were also collected for selected countries. The links to national websites accessed for data collection are presented in the Appendix. For each drug, the information regarding conditional approval or approval under exceptional circumstances was collected from the EMA's website. Then the review of Orphanet database was performed for each drug and corresponding disease targeted by the drug, what revealed that most of orphan drugs were authorized for the treatment of patients with oncologic or metabolic diseases. For that reason, additional analyzes were performed for relevant subgroups of drugs dedicated to the treatment of patients with oncology or metabolic conditions, and a comparison of the results between these 2 subgroups and drugs used for treatment of patients with other diseases (neither oncologic nor metabolic) was made. We focused on drugs for oncologic or metabolic conditions as they are large groups of orphan drugs so justify statistical analysis. Less prevalent groups could be analyzed only descriptively (EMA, 2018).

**Table 1:** Review of pricing strategies and reimbursement decision making process for orphan drugs in different European countries (Panteli et al., 2016).

**Country**	**Pricing**	**Managed entry agreements**	**Reimbursement requirements and decision-making—other remarks**
Belgium	•External reference pricing •Internal reference pricing •Value-based pricing •Negotiations	Financial arrangement	Belgium is a member of BeNeLuxA initiative (BeNeLuxA Initiative, 2018). For orphan drugs a budget impact analysis is required in the reimbursement dossier but a cost-effectiveness analysis is not. In addition, the reimbursement dossiers are not publicly available (Denis et al., 2011)(Picavet et al., 2014).
Denmark	•Internal reference pricing •Competition (retail) •Tendering (hospitals)	Financial arrangement Linked to optimizing Utilization	Reimbursement decisions are based on therapeutic effect, value added, and safety profile. In addition, the price comparisons and economic analyzes are also required in the decision-making process. The Danish Medicines Agency (a board that runs parallel to National Board of Health under the Danish Ministry of Health) decides on the reimbursement status of each drug. In addition, the Reimbursement Committee makes the recommendations and advises Danish Medicines Agency before they make any decision on whether or not to reimburse a particular drug (Møller Pedersen, 2003; Olejaz et al., 2012).
England, Scotland, Wales	•Value-based pricing •Negotiations •Profit margins	Financial arrangement, financial arrangement Linked to optimizing Utilization and primarily evidence Generation	The Rare Diseases Advisory Group exists in NHS England, NHS Scotland, NHS Wales, and NHS Northern Ireland in order to make recommendations developing and implementing the strategy for rare diseases and highly specialized services (RDAG, 2018).
France	•External reference pricing •Internal reference pricing •Value-based pricing •Negotiations	Financial arrangement	Orphan drugs undergo the same HTA, pricing, and reimbursement procedures as the other drugs (Young et al., 2017).
Germany	•External reference pricing •Internal reference pricing •Value-based pricing	Financial arrangement and financial arrangement Linked to optimizing Utilization	Orphan drugs undergo the same, pricing and reimbursement procedures as the other drugs. Benefits of particular treatments are considered proven when the drug is authorized (Young et al., 2017).
Italy	•External reference pricing •Internal reference pricing •Value-based pricing •Negotiations	Financial arrangement and financial arrangement Linked to optimizing Utilization	Orphan drugs undergo the same HTA and reimbursement procedures as the other drugs. The pricing of orphan drugs benefits from more relaxed regulations and accepted levels of uncertainty (Young et al., 2017).
Poland	•External reference pricing •Internal reference pricing •Value-based pricing •Negotiations	Financial arrangement	Orphan drugs undergo the same HTA, pricing, and reimbursement procedures as the other drugs (Tordrup et al., 2014).
Spain	•External reference pricing •Internal reference pricing	Financial arrangement and financial arrangement Linked to optimizing Utilization	Orphan drugs undergo the same HTA, pricing, and reimbursement procedures as the other drugs (Young et al., 2017).
Sweden	•Internal reference pricing •Value-based pricing •Tendering	Financial arrangement and financial arrangement Linked to optimizing Utilization	Orphan drugs undergo the same pricing and reimbursement procedures as the other drugs. The HTA process can accept more relaxed assumptions (Young et al., 2017).
The Netherlands	•External reference pricing •Internal reference pricing •Negotiations	Primarily evidence Generation	Negotiations are confidential and applied only to orphan drugs (Panteli et al., 2016).

*HTA, health technology assessment; NHS, National Health Service*.

**Table 2:** Reimbursement status of analyzed orphan drugs in selected countries.

**Medicine name**	**Belgium**	**Denmark**	**England**	**France**	**Germany**	**Italy**	**Poland**	**Scotland**	**Spain**	**Sweden**	**The netherlands**	**Wales**
Adcetris	✓	✓	✓	✗	✓	✓	✓	✓	✓	✓	✗	✓
Adempas	✓	✓	✓	✓	✓	✓	✓	✓	✓	✓	✓	✓
Alprolix	✓	✓	✓	✗	✓	✗	✗	✗	✗	✗	✓	✗
Arzerra	✗	✓	✓	✗	✓	✓	✗	✓	✓	✓	✓	✗
Atriance	✓	✓	✓	✗	✓	✓	✓	✓	✓	✗	✗	✓
Blincyto	✗	✓	✗	✗	✓	✗	✗	✓	✗	✗	✗	✓
Bosulif	✓	✓	✓	✓	✓	✓	✓	✓	✓	✓	✓	✓
Bronchitol	✗	✓	✓	✗	✓	✗	✗	✓	✗	✗	✗	✗
Carbaglu	✓	✓	✓	✓	✓	✓	✗	✓	✓	✓	✓	✗
Cayston	✓	✓	✓	✓	✓	✗	✗	✓	✓	✓	✓	✓
Ceplene	✗	✓	✗	✓	✓	✗	✗	✗	✗	✓	✗	✗
Cerdelga	✓	✓	✓	✓	✓	✗	✗	✗	✓	✗	✗	✗
Coagadex	✗	✗	✓	✗	✗	✗	✗	✗	✗	✗	✗	✗
Cometriq	✗	✓	✓	✗	✓	✗	✗	✗	✗	✓	✓	✓
Cresemba	✗	✓	✓	✓	✓	✓	✗	✓	✓	✗	✓	✓
Cystadane	✓	✓	✓	✓	✓	✗	✓	✓	✓	✓	✓	✗
Dacogen	✓	✓	✓	✗	✓	✓	✗	✗	✓	✗	✓	✗
Darzalex	✓	✓	✗	✗	✓	✗	✗	✗	✓	✗	✗	✗
Defitelio	✗	✓	✓	✗	✗	✗	✗	✓	✗	✗	✓	✓
Deltyba	✗	✗	✓	✓	✓	✗	✗	✗	✗	✗	✗	✗
Diacomit	✓	✓	✓	✓	✓	✓	✓	✓	✓	✓	✓	✓
Elaprase	✓	✓	✓	✗	✓	✓	✓	✗	✓	✗	✗	✗
Esbriet	✓	✓	✓	✓	✓	✓	✓	✓	✓	✓	✓	✗
Farydak	✓	✓	✓	✗	✓	✗	✗	✓	✗	✗	✓	✗
Firazyr	✓	✓	✓	✓	✓	✓	✓	✓	✓	✓	✗	✓
Firdapse^a^	✗	✓	✓	✓	✓	✗	✗	✓	✗	✗	✓	✗
Galafold	✓	✓	✓	✓	✓	✗	✗	✓	✗	✓	✗	✓
Gazyvaro	✓	✓	✓	✗	✓	✗	✓	✓	✓	✗	✓	✗
Gliolan	✓	✓	✗	✗	✓	✗	✗	✗	✓	✗	✗	✗
Glybera	✗	✗	✓	✗	✗	✗	✗	✗	✗	✗	✗	✗
Granupas^b^	✗	✓	✓	✓	✓	✗	✗	✗	✗	✗	✗	✗
Hetlioz	✗	✗	✗	✗	✓	✗	✗	✗	✗	✗	✗	✗
Holoclar	✓	✓	✓	✗	✗	✗	✗	✗	✗	✗	✓	✗
Iclusig	✓	✓	✓	✓	✓	✓	✗	✓	✓	✓	✓	✓
Idelvion	✓	✓	✓	✗	✗	✗	✗	✗	✗	✗	✓	✗
Imbruvica	✓	✓	✓	✓	✓	✓	✓	✓	✓	✓	✓	✓
Imnovid^c^	✓	✓	✓	✓	✓	✓	✗	✓	✓	✓	✓	✓
Increlex	✓	✓	✓	✓	✓	✓	✓	✓	✓	✓	✓	✓
Inovelon	✓	✓	✗	✓	✓	✓	✗	✓	✓	✓	✓	✓
Kalydeco	✓	✓	✓	✓	✗	✓	✗	✗	✓	✗	✓	✓
Kanuma	✗	✓	✓	✗	✗	✗	✗	✗	✗	✗	✗	✗
Ketoconazole HRA	✗	✓	✓	✓	✓	✗	✗	✗	✗	✓	✗	✗
Kolbam	✗	✗	✓	✗	✗	✗	✗	✗	✗	✗	✗	✗
Kuvan	✓	✓	✓	✓	✓	✓	✗	✗	✓	✗	✓	✗
Kyprolis	✓	✓	✗	✗	✓	✓	✗	✗	✓	✗	✗	✗
Lartruvo	✗	✓	✗	✗	✓	✗	✗	✗	✓	✗	✗	✗
Lenvima	✗	✓	✗	✓	✓	✓	✗	✓	✓	✗	✓	✗
Lynparza	✓	✓	✓	✗	✓	✓	✓	✓	✓	✗	✓	✗
Mepact	✗	✓	✗	✓	✓	✓	✗	✓	✓	✗	✗	✗
Mozobil	✓	✓	✓	✓	✓	✓	✓	✓	✓	✗	✗	✓
Nexavar	✓	✓	✓	✓	✓	✓	✓	✓	✓	✗	✓	✓
NexoBrid	✓	✓	✗	✗	✓	✓	✗	✗	✗	✗	✗	✗
Ninlaro	✓	✓	✓	✗	✓	✗	✗	✗	✗	✗	✗	✗
Nplate	✓	✓	✗	✓	✓	✓	✗	✓	✓	✗	✓	✗
Ocaliva	✗	✓	✓	✓	✓	✗	✗	✓	✗	✗	✗	✗
Ofev	✓	✓	✓	✓	✓	✓	✗	✓	✓	✗	✓	✗
Onivyde	✗	✓	✗	✗	✓	✗	✗	✗	✗	✗	✗	✗
Opsumit	✓	✓	✓	✓	✓	✓	✓	✓	✓	✓	✓	✓
Orphacol	✗	✓	✓	✓	✓	✗	✗	✗	✓	✗	✓	✗
Peyona^d^	✗	✓	✗	✗	✓	✗	✗	✗	✓	✗	✗	✗
Plenadren	✗	✓	✗	✗	✓	✓	✗	✓	✗	✗	✗	✗
Procysbi	✗	✓	✓	✓	✓	✗	✗	✗	✗	✗	✗	✗
Ravicti	✗	✓	✓	✗	✓	✗	✗	✗	✗	✗	✗	✗
Raxone	✗	✓	✓	✗	✓	✗	✗	✓	✗	✓	✓	✗
Revestive	✗	✓	✓	✓	✓	✗	✗	✗	✓	✗	✗	✗
Revlimid	✓	✓	✓	✓	✓	✓	✓	✓	✓	✓	✓	✓
Scenesse	✗	✗	✗	✗	✗	✗	✗	✗	✗	✗	✗	✗
Signifor	✗	✓	✗	✓	✓	✓	✗	✓	✓	✓	✓	✓
Siklos	✓	✗	✓	✓	✓	✗	✗	✓	✓	✗	✓	✗
Sirturo	✗	✓	✓	✗	✓	✓	✗	✗	✗	✗	✓	✓
Soliris	✓	✓	✓	✗	✓	✓	✗	✗	✓	✗	✗	✓
Sprycel	✓	✓	✓	✓	✓	✓	✓	✓	✓	✓	✓	✓
Strensiq	✗	✓	✓	✗	✗	✗	✗	✗	✗	✗	✗	✗
Strimvelis	✗	✗	✗	✗	✗	✗	✗	✗	✗	✗	✓	✗
Sylvant	✗	✓	✗	✗	✓	✓	✗	✗	✓	✗	✓	✗
Tasigna	✓	✓	✓	✓	✓	✓	✓	✓	✓	✓	✓	✗
Tepadina	✓	✓	✗	✗	✓	✓	✓	✗	✓	✗	✗	✗
Thalidomide Celgene^e^	✓	✓	✓	✓	✓	✗	✗	✗	✗	✓	✓	✓
Tobi Podhaler	✓	✓	✓	✓	✓	✗	✗	✓	✗	✗	✓	✗
Torisel	✓	✓	✓	✗	✓	✓	✓	✗	✓	✗	✗	✗
Translarna	✗	✓	✓	✗	✗	✗	✗	✗	✗	✗	✗	✓
Unituxin	✗	✗	✗	✗	✓	✗	✗	✗	✗	✗	✗	✗
Venclyxto	✗	✓	✗	✗	✓	✗	✗	✗	✗	✗	✓	✗
Vidaza	✓	✓	✓	✓	✓	✓	✓	✓	✓	✗	✓	✗
Vimizim	✗	✓	✓	✗	✓	✓	✗	✗	✗	✗	✗	✓
Volibris	✓	✓	✓	✓	✓	✓	✓	✓	✓	✓	✓	✓
Votubia	✓	✓	✓	✓	✓	✓	✓	✗	✓	✓	✓	✗
Vpriv	✓	✓	✓	✓	✓	✓	✓	✓	✓	✗	✓	✓
Vyndaqel	✓	✓	✗	✓	✗	✓	✗	✗	✓	✗	✓	✗
Wakix	✗	✗	✗	✓	✓	✗	✗	✗	✗	✗	✗	✗
Xagrid	✓	✓	✓	✓	✓	✓	✓	✓	✓	✓	✓	✓
Xaluprine^f^	✗	✓	✗	✓	✓	✗	✗	✓	✓	✓	✓	✓
Yondelis	✓	✓	✓	✗	✓	✓	✓	✗	✓	✗	✗	✗
Zalmoxis	✗	✗	✗	✗	✗	✗	✗	✗	✗	✗	✗	✗
Zavesca	✓	✓	✓	✓	✓	✓	✗	✓	✓	✓	✓	✗
number of positive decisions	54	84	69	50	81	47	26	47	56	30	52	29
number of negative decisions	41	11	26	45	14	48	69	48	39	65	43	66

*✓, reimbursement; ✗, no reimbursement*.

a *Previously Zenas*.

b *Previously Para-aminosalicylic acid Lucane*.

c *Previously Pomalidomide Celgene*.

d *Previously Nymusa*.

e *Previously Thalidomide Pharmion*.

f *Previously Mercaptopurine Nova Laboratories*.

Significant differences between reimbursement systems among the countries can impact the comparisons and agreement in recommendations and reimbursement status for the analyzed drugs. The agreement between recommendations and reimbursement decisions for each country separately as well as between countries were assessed using the κ coefficient for measurement of agreement, with values lower than 0 denoting less than chance agreement; between 0.01 and 0.20, slight agreement; between 0.21 and 0.40, fair agreement; between 0.41 and 0.60, moderate agreement; between 0.61 and 0.80, substantial agreement; and between 0.81 and 0.99, almost perfect agreement (Viera and Garrett, 2005). All κ coefficients were supported with 95% confidence intervals (CIs) and rounded to 2 decimal places.

The comparison of 2 nominal variables was performed using the χ^2^-test or the Fisher exact test where applicable, depending on expected cell counts in contingency tables. The results of the tests were presented as *p-*values rounded to 4 decimal places. The data were summarized with counts and percentages.

The impact of the EMA's conditional approval as well as approval under exceptional circumstances was assessed using the logistic regression and presented as odds ratio (OR) showing the odds for reimbursement when these types of approval were granted compared with no conditional approval or approval under exceptional circumstances status. Logistic regression was also used to investigate the impact of type of the disease on the type of approval. All ORs were presented with 95% CI rounded to 2 decimal places and corresponding *p-*values rounded to 4 decimal places. A *p*-value of < 0.05 was considered statistically significant. Statistical analyzes were carried out in the JMP® software, version 13.1.0 (SAS Institute Inc., 2016, Cary, North Carolina 27513, USA).

## Results

### Analysis of reimbursement decisions for orphans in selected countries

**Figure 1:**
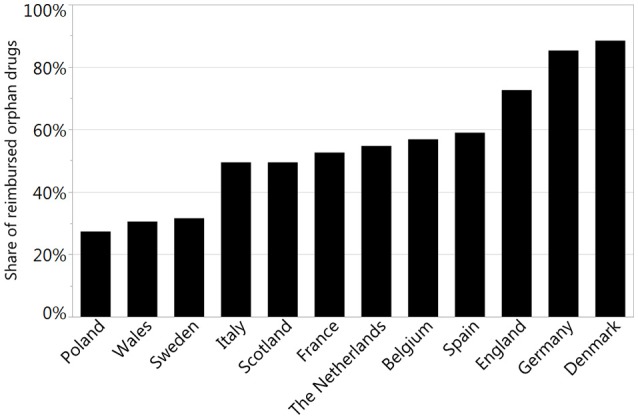
The percentage of reimbursed orphan drugs in selected countries.

The reimbursement status was assessed for a total of 95 orphan drugs in 12 countries. The percentage of reimbursed drugs varied from 27% in Poland to 88% in Denmark (Figure 1). Considering the type of a disease (metabolic/oncologic) a statistically significant relation with the reimbursement status was observed only in Germany (Table 3). Regarding the reimbursement status, the highest, substantial agreement was observed between Spain and Italy, and the lowest agreement was detected between Germany and England, with κ of 0.64 and 0.01, respectively (Table 4).

**Table 3:** The relation in reimbursement decisions and type of a disease in selected countries.

**Country**	**Reimbursement**	**Metabolic diseases**	**Oncologic diseases**	**Other diseases**	**Total**	***p*-value (χ^2^-test)**
Belgium	Not reimbursed	12 (54.55%)	12 (33.33%)	17 (45.95%)	41	0.2597
	Reimbursed	10 (45.45%)	24 (66.67%)	20 (54.05%)	54
Denmark	Not reimbursed	4 (18.18%)	2 (5.56%)	5 (13.51%)	11	0.2881
	Reimbursed	18 (81.82%)	34 (94.44%)	32 (86.49%)	84
England	Not reimbursed	3 (13.64%)	14 (38.89%)	9 (24.32%)	26	0.0971
	Reimbursed	19 (86.36%)	22 (61.11%)	28 (75.68%)	69
France	Not reimbursed	10 (45.45%)	21 (58.33%)	14 (37.84%)	45	0.2105
	Reimbursed	12 (54.55%)	15 (41.67%)	23 (62.16%)	50
Germany	Not reimbursed	7 (31.82%)	1 (2.78%)	6 (16.22%)	14	0.0097^*^
	Reimbursed	15 (68.18%)	35 (97.22%)	31 (83.78%)	81
Italy	Not reimbursed	12 (54.55%)	15 (41.67%)	21 (56.76%)	48	0.3971
	Reimbursed	10 (45.45%)	21 (58.33%)	16 (43.24%)	47
Poland	Not reimbursed	18 (81.82%)	21 (58.33%)	30 (81.08%)	69	0.0507
	Reimbursed	4 (18.18%)	15 (41.67%)	7 (18.92%)	26
Scotland	Not reimbursed	14 (63.64%)	17 (47.22%)	17 (45.95%)	48	0.3715
	Reimbursed	8 (36.36%)	19 (52.78%)	20 (54.05%)	47
Spain	Not reimbursed	11 (50.00%)	10 (27.78%)	18 (48.65%)	39	0.1205
	Reimbursed	11 (50.00%)	26 (72.22%)	19 (51.35%)	56
Sweden	Not reimbursed	15 (68.18%)	22 (61.11%)	28 (75.68%)	65	0.4082
	Reimbursed	7 (31.82%)	14 (38.89%)	9 (24.32%)	30
The Netherlands	Not reimbursed	13 (59.09%)	16 (44.44%)	14 (37.84%)	43	0.2821
	Reimbursed	9 (40.91%)	20 (55.56%)	23 (62.16%)	52
Wales	Not reimbursed	16 (72.73%)	26 (72.22%)	24 (64.86%)	66	0.7376
	Reimbursed	6 (27.27%)	10 (27.78%)	13 (35.14%)	29
**Total**	**22**	**36**	**37**	**95**

* *statistically significant*.

**Table 4:** The agreement in reimbursement decisions between selected countries.

**Country**	**Denmark**	**England**	**France**	**Germany**	**Italy**	**Poland**	**Scotland**	**Spain**	**Sweden**	**The netherlands**	**Wales**
Belgium	0.25 (0.10 to 0.40)	0.30 (0.12 to 0.49)	0.28 (0.09 to 0.47)	0.18 (0.02 to 0.35)	0.47 (0.30 to 0.65)	0.44 (0.30 to 0.59)	0.31 (0.12 to 0.50)	0.57 (0.40 to 0.74)	0.24 (0.07 to 0.41)	0.36 (0.17 to 0.55)	0.14 (0.03 to 0.31)
Denmark		0.19 (−0.01 to 0.40)	0.12 (−0.01 to 0.26)	0.40 (0.14 to 0.67)	0.23 (0.10 to 0.35)	0.09 (0.03 to 0.16)	0.19 (0.06 to 0.31)	0.27 (0.11 to 0.42)	0.11 (0.04 to 0.19)	0.18 (0.04 to 0.32)	0.11 (0.04 to 0.18)
England			0.20 (0.02 to 0.38)	**0.01** (–**0.18 to 0.20)**	0.08 (−0.10 to 0.26)	0.21 (0.10 to 0.33)	0.20 (0.03 to 0.38)	0.06 (−0.13 to 0.25)	0.15 (0.02 to 0.28)	0.23 (0.05 to 0.41)	0.14 (0.01 to 0.27)
France				0.23 (0.09 to 0.38)	0.26 (0.07 to 0.46)	0.18 (0.01 to 0.35)	0.47 (0.30 to 0.65)	0.40 (0.22 to 0.59)	0.42 (0.26 to 0.59)	0.41 (0.22 to 0.59)	0.20 (0.02 to 0.37)
Germany					0.21 (0.07 to 0.34)	0.12 (0.05 to 0.19)	0.25 (0.11 to 0.38)	0.30 (0.14 to 0.47)	0.15 (0.06 to 0.23)	0.08 (−0.08 to 0.23)	0.04 (−0.05 to 0.14)
Italy						0.43 (0.27 to 0.59)	0.37 (0.18 to 0.56)	**0.64 (0.49 to 0.79)**	0.22 (0.03 to 0.40)	0.31 (0.12 to 0.50)	0.28 (0.10 to 0.46)
Poland							0.34 (0.17 to 0.51)	0.42 (0.28 to 0.56)	0.29 (0.09 to 0.50)	0.15 (−0.01 to 0.32)	0.16 (−0.05 to 0.36)
Scotland								0.39 (0.21 to 0.57)	0.43 (0.26 to 0.60)	0.47 (0.30 to 0.65)	0.32 (0.15 to 0.50)
Spain									0.25 (0.09 to 0.41)	0.36 (0.17 to 0.55)	0.15 (−0.01 to 0.32)
Sweden										0.35 (0.18 to 0.51)	0.34 (0.13 to 0.54)
The Netherlands											0.17 (−0.01 to 0.34)

*κ coefficients with 95% confidence intervals. The lowest (Germany and England) and the highest (Italy and Spain) values are in bold*.

### The impact of conditional approval and approval under exceptional circumstances on reimbursement status

The conditional approval was associated with reimbursement status only in France, Italy, and Spain. The EMA's conditional approval status in France decreased odds for reimbursement by 80% (OR, 0.20; 95% CI, 0.05–0.76; *p* = 0.0185), in Italy by 77% (OR, 0.23, 95% CI, 0.06–0.88; *p* = 0.0324), and in Spain by 78% (OR, 0.22; 95% CI, 0.06–0.77; *p* = 0.0182) (Table 5). Approval under exceptional circumstances was associated with the reimbursement status only in Germany, where the odds for reimbursement were 85% (OR, 0.15; CI 95%, 0.04–0.53; *p* = 0.0034) lower for drugs approved under exceptional circumstances when compared with other drugs (Table 6).

**Table 5:** Relation between reimbursement status and conditional approval.

**Country**	**Conditional approval**				***p*-value (χ^2^-test)**
	**No**		**Yes**	
	**Not reimbursed**	**Reimbursed**	**Not reimbursed**	**Reimbursed**	
Belgium	32 (39.51%)	49 (60.49%)	9 (64.29%)	5 (35.71%)	0.0839
Denmark	9 (11.11%)	72 (88.89%)	2 (14.29%)	12 (85.71%)	0.7318
England	21 (25.93%)	60 (74.07%)	5 (35.71%)	9 (64.29%)	0.4481
France	34 (41.98%)	47 (58.02%)	11 (78.57%)	3 (21.43%)	0.0113^*^
Germany	11 (13.58%)	70 (86.42%)	3 (21.43%)	11 (78.57%)	0.4443
Italy	37 (45.68%)	44 (54.32%)	11 (78.57%)	3 (21.43%)	0.0230^*^
Poland	57 (70.37%)	24 (29.63%)	12 (85.71%)	2 (14.29%)	0.2344
Scotland	38 (46.91%)	43 (53.09%)	10 (71.43%)	4 (28.57%)	0.0903
Spain	29 (35.80%)	52 (64.20%)	10 (71.43%)	4 (28.57%)	0.0123^*^
Sweden	54 (66.67%)	27 (33.33%)	11 (78.57%)	3 (21.43%)	0.3762
The Netherlands	34 (41.98%)	47 (58.02%)	9 (64.29%)	5 (35.71%)	0.1215
Wales	57 (70.37%)	24 (29.63%)	9 (64.29%)	5 (35.71%)	0.6480

* *, Bold values—Statistically significant*.

**Table 6:** Relation between reimbursement status and approval under exceptional circumstances.

**Country**	**Approval under exceptional circumstances**				***p*-value (χ^2^-test)**
	**No**		**Yes**	
	**Not reimbursed**	**Reimbursed**	**Not reimbursed**	**Reimbursed**
Belgium	32 (39.51%)	49 (60.49%)	9 (64.29%)	5 (35.71%)	0.0839
Denmark	8 (9.88%)	73 (90.12%)	3 (21.43%)	11 (78.57%)	0.2123
England	23 (28.40%)	58 (71.60%)	3 (21.43%)	11 (78.57%)	0.5893
France	37 (45.68%)	44 (54.32%)	8 (57.14%)	6 (42.86%)	0.4276
Germany	8 (9.88%)	73 (90.12%)	6 (42.86%)	8 (57.14%)	0.0013^*^
Italy	39 (48.15%)	42 (51.85%)	9 (64.29%)	5 (35.71%)	0.2648
Poland	58 (71.60%)	23 (28.40%)	11 (78.57%)	3 (21.43%)	0.5893
Scotland	40 (49.38%)	41 (50.62%)	8 (57.14%)	6 (42.86%)	0.5919
Spain	31 (38.27%)	50 (61.73%)	8 (57.14%)	6 (42.86%)	0.1850
Sweden	55 (67.90%)	26 (32.10%)	10 (71.43%)	4 (28.57%)	0.7932
The Netherlands	36 (44.44%)	45 (55.56%)	7 (50.00%)	7 (50.00%)	0.6998
Wales	56 (69.14%)	25 (30.86%)	10 (71.43%)	4 (28.57%)	0.8634

* *, Bold values—Statistically significant*.

### The impact of type of disease on conditional approval and approval under exceptional circumstances

Out of all drugs, 36 (38%) were used for treatment of patients with oncologic diseases (e.g., relapsed or refractory CD30+ Hodgkin lymphoma), 22 (23%) for metabolic diseases (e.g., type 1 Gaucher disease), and 37 (39%) for other diseases (e.g., cystic fibrosis, severe hepatic veno-occlusive disease). Both conditional approval and approval under exceptional circumstances were associated with the type of the disease. Almost one-third of orphan drugs for metabolic diseases were granted approval under the exceptional circumstances compared with only 6% in the case of drugs for oncologic diseases; however, in the case of conditional approval the situation was reversed: a quarter of orphan drugs for oncologic diseases was approved conditionally, compared with 0% of orphan drugs for metabolic diseases (Table 7).

**Table 7:** Relation between conditional approval, approval under exceptional circumstances, and type of disease.

**Disease type**	**Conditional approval**		***p*-value**	**Approval under exceptional circumstances**		***p*-value (χ^2^-test)**
	**No**	**Yes**		**No**	**Yes**
Oncologic	27 (75%)	9 (25%)	0.0323^*^	34 (94.44%)	2 (5.56%)	0.0227^*^
Metabolic	22 (100%)	0 (0%)		15 (68.18%)	7 (31.82%)
Other	32 (86.49%)	5 (13.51%)		32 (86.49%)	5 (13.51%)

* *Statistically significant*.

Drugs for metabolic diseases were 8.25-fold (95% CI, 1.6–46.90; *p* = 0.0123) more likely to be approved under exceptional circumstances, but had 96% less odds for being conditionally approved (OR, 0.04; 95% CI, 0.00006–0.67; *p* = 0.0092) when compared to other drugs for non-metabolic and non-oncologic diseases. The opposite was observed for drugs used in treatment of patients with oncologic diseases. Those drugs were 87% less likely to be approved under exceptional circumstances (OR, 0.83; 95% CI, 0.01–0.84; *p* = 0.0301) and had the odds for being conditionally approved increased 10-fold (95% CI, 1.58–287.77; *p* = 0.006) when compared with other drugs for non-metabolic and non-oncologic diseases.

### Additional analysis of recommendations for orphans in selected countries

**Figure 2:**
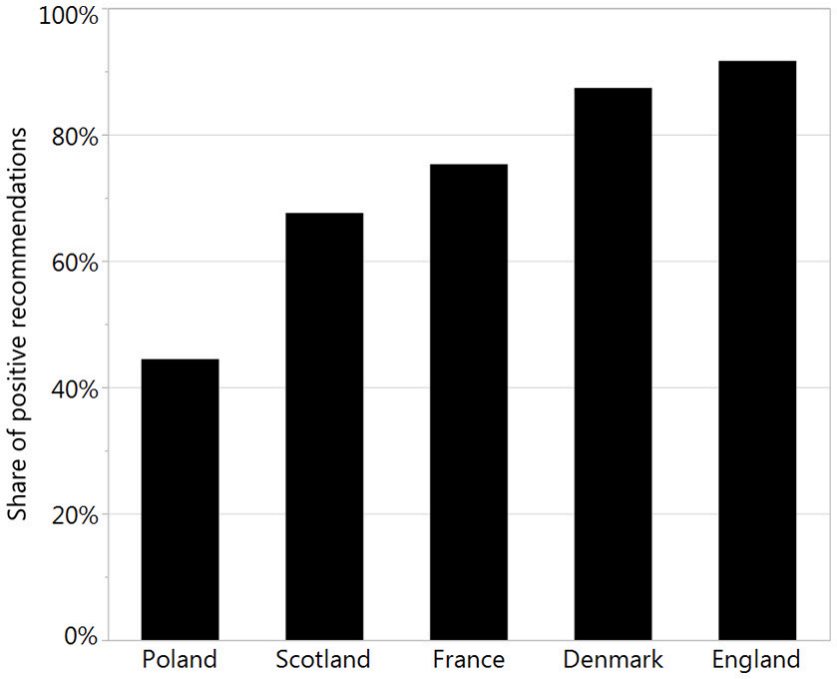
The percentage of positive reimbursement recommendations.

To perform a sophisticated analysis of recommendations, we made a review of officially available websites and databases and collected relevant data. We found all necessary data only for 5 countries: Denmark, England, France, Poland, and Scotland. The percentage of positive recommendations varied from 44% in Poland to 92% in England (Figure 2).

The agreement in recommendation type (negative or positive) was assessed between Denmark, England, France, Poland, and Scotland. For these countries information about positive and negative recommendations was available online. The highest agreement was observed between England and Scotland (κ of 0.54) and the lowest between England and Denmark (insignificant κ of −0.04) (Table 8).

**Table 8:** The agreement in reimbursement recommendations between selected countries.

**Country**	**England**	**France**	**Poland**	**Scotland**
Denmark	−0.04 (−0.11 to 0.02)	0.13 (−0.08 to 0.33)	0.00 (0.00 to 0.00)	0.06 (−0.05 to 0.17)
England		0.32 (−0.08 to 0.71)	0.17 (−0.05 to 0.40)	0.54 (0.16 to 0.91)
France			0.16 (−0.07 to 0.39)	0.12 (−0.11 to 0.35)
Poland				0.27 (0.03 to 0.51)

*κ coefficients with 95% confidence intervals*.

**Figure 3:**
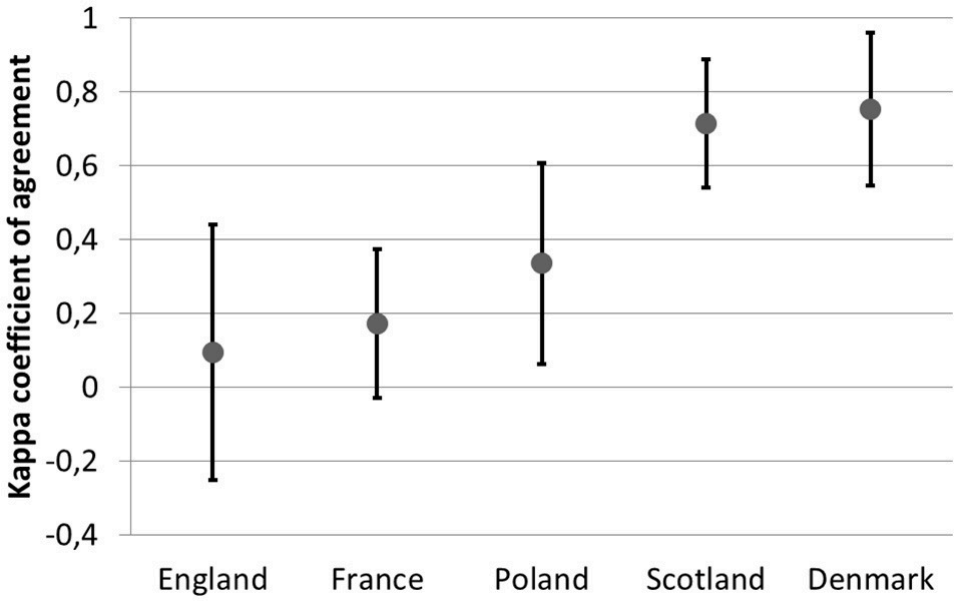
The agreement between recommendations and reimbursement status in the analyzed countries.

The observed agreement between recommendation and the reimbursement status within countries varied from 0.09 (−0.25 to 0.44) in England to 0.7 (0.55–0.96) in Denmark (Figure 3).

## Discussion

The study investigated the shares of reimbursed orphan drugs among all those with orphan designation among several EU countries and the agreement between them. In addition, to our best knowledge this is the first study that investigates the impact of conditional approval and exceptional circumstances on reimbursement decisions in EU countries.

In France, Italy, and Spain, conditional approval significantly decreased the odds for reimbursement, which suggest that the decision-making body in those countries waits for further data on efficacy or safety (the EMA, after providing conditional approval, requires the applicant to provide further data within the agreed time frame) while in other considered countries the impact of approval status was assessed although not significant. However, a similar association was observed in terms of approval under exceptional circumstances in Germany with the same impact. The observed agreement in reimbursement decisions among the countries varied from agreement on a random level to substantial agreement.

The reimbursement status was significantly associated with the type of the disease (metabolic or oncologic) only for Germany (*p* < 0.01). Taking into account all considered countries the type of disease was however significantly associated with the type of authorization by EMA—drugs for metabolic diseases were 8.25-fold more likely to be approved under exceptional circumstances, oncologic drugs had the odds for being conditionally approved increased 10-fold.

In response to increasing health expenditures, more and more third-party payers tend to rationalize their expenses by implementing cost-effectiveness criterion prior to pricing and reimbursement decisions or at least by referencing pharmaceutical prices of the countries in which evidence on cost-effectiveness are mandated. Objective decision-making for public reimbursement has to be based on clinical and economic criteria, but social issues should also be considered. It is important to note that this study analyzed mostly old (pre-2004) member states of the European Union (EU15) and only one post-2004 accession state (Poland). This may raise some concerns because the comparison was between high-income and low-income countries. However, the results of this study provide a good basis for further research on this subject, especially that the Central Eastern European region has expanded its pharmaceutical share of health spending at an 8-fold higher annual rate compared with EU15 (Jakovljevic et al., 2016). This may result in a faster increase in the share of reimbursed orphan drugs in post-2004 member states. This trend might be then enhanced by new generic versions of orphan drugs that should enter the pharmaceutical market shortly, because patent protection and the exclusivity period for several orphan drugs will expire soon. However, this could raise some concerns as this substitution should be based not only on an economic analysis but also on clinical and patient-reported outcomes (Di Paolo and Arrigoni, 2018). This is particularly important because orphan drugs together with targeted biologics are considered the most expensive types of pharmaceuticals (Jakovljevic and Yamada, 2017).

Financial aspects are the major determinant of the observed differences among the analyzed countries. Many countries apply additional mechanisms to allow access to medicines for which there is high uncertainty (at the time of marketing authorization) regarding effectiveness, cost-effectiveness, or budget impact. These mechanisms are commonly referred to as managed entry agreements (MEAs), that is, negotiations between payers and manufacturers to share the cost of uncertainty. The objective of MEAs is to facilitate access to new and expensive medicines, including orphan drugs.

Various approaches to pricing are another factor that contributed to differences in the availability of orphan drugs. Price revisions are conducted periodically or when necessary, and this varies between countries. In Belgium and Germany, regular revisions may concern only some groups of drugs. Those revisions could be associated with negotiations between the payer and marketing authorization holder (as in France or Italy) or with the planned review of reimbursement decisions after a specific fixed period (as in Poland or the Netherlands) (Panteli et al., 2016).

Drugs for metabolic diseases were more likely to be approved under exceptional circumstances but less likely to be conditionally approved compared with other orphan drugs. This was due to inability to collect comprehensive data on their efficacy and safety. Many drugs for metabolic diseases come as enzyme-replacement therapies, the approval of which may not require comprehensive data on safety. On the other hand, oncologic orphan drugs were more often approved under conditional marketing authorization to provide patients with new drugs as fast as possible even if clinical data were immature or incomplete. However, marketing authorizations holders are obliged to provide relevant data within a defined period to maintain the registration status.

We performed a review on publications for current reimbursement policies in European settings, however no other studies investigated the influence of authorization details on the reimbursement. We reviewed some papers published lately that face the problems. The present study is innovative, as we did not identify valid studies on the similar topic carried out for European countries. A study by Kawalec et al. (2016) had a similar approach, but a revision of methods and an update of input data were necessary to provide the topical and valid review on the management of orphan drugs in the selected European countries. The study revealed that 21% of EMA-authorized orphan drugs were reimbursed in 8 European countries that were studied: 49% of those orphan drugs had positive reimbursement recommendations, 54% of those had conditional reimbursement recommendations, and 16% had negative reimbursement recommendations. The shares of the orphan drugs for oncologic diseases, orphan drugs for ultrarare diseases and other orphan drugs that were assessed by HTA agencies were similar, with the lowest percentage observed in ultra-orphan drugs (72%) and the highest in other orphan drugs (80%). While the highest rate of reimbursement was observed among drugs with positive or conditional recommendation, a high rate of reimbursement (11%) was revealed among ultra-orphan drugs that had never been assessed by any HTA agency (Kawalec et al., 2016). Although methods used in the previous study by Kawalec et al. (2016) seem quite similar, the results are unsuitable for direct comparisons. The orphan drugs varied between this study and the previous one, since in the period between the 2 studies some new drugs were approved as orphans and some drugs failed to maintain the status of orphan drugs. Consequently, a dataset on orphan drugs differed significantly between the previous and present study, which influenced the results and conclusions. In 2015, the study focused on correlations between recommendations and reimbursement decisions in selected countries, while present study focused on the odds of agreement between countries and the impact of special EMA approval modes (conditional approval and approval under exceptional circumstances) on chances for reimbursement.

The percentages of reimbursed orphan drugs were lower in the present study than those observed in other studies (Garau and Mestre-Ferrandiz, 2009; Gammie et al., 2015); as we consider the revealed differences were due different sets of orphan drugs taken under consideration because 3 years ago partly another set of orphan drugs was analyzed. That is why we observed 32% of reimbursed orphan drugs compared to 69% for Sweden however for Scotland the observed percentage of reimbursed orphans 49% was similar to reported 54%.

A comparative analysis on the access to orphan drugs in a sample of Balkan countries −5 EU member states (Bulgaria, Croatia, Greece, Romania, Slovenia) and 2 EU candidate countries (Serbia, Montenegro)—was carried out by Pejcic et al. (2018). It revealed significant inequalities among these countries as well as a substantial lack of access to orphan drugs approved for EU market and a need for improvement in accessibility of orphan drugs in the Balkan states.

Another study was carried out by Sarnola et al. (2018) to assess reimbursement and pricing policies specific to orphan medicines and the availability and distribution settings of 10 recently authorized medicinal products in 24 European countries. No specific policies were implemented in the assessment of reimbursement status of orphan drugs in 22 countries, and in 20 countries no special policies were implemented for pricing. Moreover, the availability of orphan products varied between countries. The authors emphasize the importance of discussing if orphan drugs should be placed in separated group for specific reimbursement regulations to facilitate patient access.

Adkins et al. (2017) made a review to evaluate different mechanisms that have been introduced to facilitate patient access to oncologic orphan drugs in 5 different countries (Australia, Canada, England, France, and Sweden), using 8 oncologic orphan drugs and non-orphan oncologic drugs as examples of their application. It was revealed that additional assessment processes were rarely used and decisions were mostly driven by proving cost-effectiveness using standard incremental cost-effectiveness ratio thresholds. Application of standard HTA criteria to oncologic orphan drugs in many countries does not consider any specificity clinical and cost input producing high cost-effectiveness results (above standard cost-effectiveness thresholds) and HTA agencies should adopt a more flexible approach to cost-effectiveness, considering high unmet medical needs, limited clinical effectiveness evidence but also the small patient numbers involved in therapy with orphan drugs.

A review by Zelei et al. (2016) focused on potentially relevant value drivers in the reimbursement process of orphan drugs. Due to external price referencing of pharmaceuticals, the relative budget impact of orphan drugs is expected to be higher in CEE than in Western European countries unless accessibility of patients remains more limited in poorer European regions. Good clinical evidence seems to play a fundamental role providing an evidence for clinical effectiveness but also input to cost-effectiveness analyzes, which play a key role in decision-making in these countries.

There were substantial differences in the total public expenditure on orphan drugs per capita in participant countries. The absolute spending was clearly associated with the economic status of the countries. The generalizability of the findings may be limited due to several reasons. It should be emphasized that the orphan status of medicines is flexible and can change over time, which considerably influenced the conclusions from the present study compared with the previous results (Orphanet, 2018).

Although our study was planned and conducted so that it was as reliable as possible, it is not free from limitations. First of all, not all European countries were considered in the study and this may introduce some selection bias. We considered only those countries for which the required data were available online. The results should be interpreted in the context of analyzed countries and may not be generalizable to the EU as a whole; however, the results can be generalizable to other potential orphan drugs, which constitute the evident strength of the study. In addition, the differences in decision-making processes between the analyzed countries resulted in the lack of data for recommendations for some of them.

Additionally, collection of data from different websites is prone to errors hence there is a great need of the unified system to bring together relevant data for reimbursed drugs for all EU member states.

The κ coefficient with 95% CI was used to analyze the agreement in reimbursement statuses between countries as well as the agreement between reimbursement recommendations and statuses within countries. To our knowledge, this is the best approach; however, it could be influenced by the presence of bias between countries and by the distributions of reimbursement statuses. Hence, the presented coefficients should be treated as descriptive statistics rather than an inference. The agreement as well as predictive abilities of conditional approval and approval under exceptional circumstances could be confounded by other factors that were not analyzed in this study such as results of economic analyzes, reliability of clinical trials of specific drugs, or experts' opinions.

Despite some limitations the study have several strengths: a comprehensive analysis for eligible countries with different reimbursement systems was performed, considering all orphan drugs approved in the EU; it's a novelty as no such studies were conducted before. Results of the study would be useful for reimbursement decision making and orphan drug policies in European countries as international comparisons and review of reimbursement statuses in other states could be an important aspect providing simpler and faster evaluation of orphan drug value. The results of this study should constitute a good basis for further research.

## Conclusions

The percentage of reimbursed orphan drugs varied among the countries and was the lowest in Poland and the highest in Denmark. The highest, substantial agreement in reimbursement decisions was observed between Italy and Spain, and the highest agreement in recommendations was observed between England and Scotland. The conditional approval significantly decreased the chance for reimbursement in France, Italy, and Spain. The approval granted under the exceptional circumstances had the same impact only in Germany. Drugs for metabolic diseases were more likely to be approved under exceptional circumstances, but had lesser odds for being conditionally approved when compared to other drugs for non-metabolic and non-oncologic diseases. The opposite was observed for drugs used in treatment of patients with oncologic diseases.

## Author contributions

PK developed the concept, designed and coordinated the study. PK and KM analyzed the data and wrote the draft of the manuscript. PK, WT, CS, and AP wrote the final version of the manuscript.

### Conflict of interest statement

The authors declare that the research was conducted in the absence of any commercial or financial relationships that could be construed as a potential conflict of interest.

## Appendix—list of reference websites

Poland (http://www.mz.gov.pl, http://www.aotm.gov.pl)

England (https://www.england.nhs.uk, https://www.nice.org.uk)

Scotland (http://www.isdscotland.org, https://www.scottishmedicines.org.uk)

Sweden (https://www.tlv.se)

Wales (www.wales.nhs.uk, http://www.awmsg.org)

Germany (https://www.dimdi.de, https://www.g-ba.de)

France (http://solidarites-sante.gouv.fr, https://www.has-sante.fr)

The Netherlands (https://www.gipdatabank.nl)

Denmark (http://www.medicinpriser.dk, https://laegemiddelstyrelsen.dk)

Belgium (http://www.inami.fgov.be)

Spain (https://www.vademecum.es)

Italy (http://www.aifa.gov.it)
